# Comment on Laratta et al. Marital Stability and Quality of Couple Relationships after Acquired Brain Injury: A Two-Year Follow-Up Clinical Study. *Healthcare* 2021, *9*, 283

**DOI:** 10.3390/healthcare9081024

**Published:** 2021-08-10

**Authors:** Brenda van den Broek, Boudewijn Bus, Sophie Rijnen

**Affiliations:** 1 Multidisciplinary Specialist Centre for Brain Injury and Neuropsychiatry, GGZ Oost Brabant, Kluisstraat 2, 5427 EM Boekel, The Netherlands; baa.bus@ggzoostbrabant.nl (B.B.); sjm.rijnen@ggzoostbrabant.nl (S.R.); 2 School for Mental Health and Neuroscience, Maastricht University Medical Centre, Universiteitssingel 40, 6229 ER Maastricht, The Netherlands; 3 Limburg Brain Injury Center, Maastricht University, 6200 MD Maastricht, The Netherlands

We read with great interest the recent article by Laratta et al. [1] entitled ‘*Marital Stability and Quality of Couple Relationships after Acquired Brain Injury: A Two-Year Follow-Up Clinical Study*’. The authors identify several demographic and clinical factors that are related to the quality of couple relationships following acquired brain injury (ABI). More specifically, they employ regression analyses to determine which factors predict (1) the functioning of couples as measured by the Dyadic Adjustment Scale (DAS [2]) and (2) the quality of the family relationship as measured by the Family Relationship Index (FRI [3]). Results showed that the DAS and FRI values of patients were predicted by educational level whereby a higher educational level was associated with better relationship and family functioning. DAS values of partners were predicted by etiology whereby partners of traumatic brain injury patients reported lower couple functioning than partners of patients with vascular injuries. The FRI values of partners were predicted by religion commitment; partners who spent more time on religious activities reported a better quality of family relationships.

The article’s focus is highly relevant given the often drastic effects of ABI on romantic relationships [4,5] and the relatively small number of existing studies that have aimed to detect which factors affect relationship quality after ABI. We, furthermore, appreciate that the authors included both patients and partners in their study, as the inclusion of both perspectives is often lacking in quantitative research on partner relationships following ABI [4].

However, given the relatively small sample size (*n* = 35), and the rather large number of predictors (11) in the regression analyses, the study’s analyses may have been underpowered. Using the G*Power software [6] to calculate the desired sample size to achieve a power of 0.8 for a multiple linear regression model with 11 predictors when *a* is set at 0.05 showed that a sample size of 123 would be needed to detect medium effects (*f^2^* = 0.15). Consequently, with the current approach, one might incorrectly assume that (some of the) factors included in the study are unrelated to relationship quality, while in fact they may have failed to reach statistical significance only because of the lack of power.

Although it was probably not an option for Laratta et al. [1], given their limited sample size and their focus on the demographic and clinical variables, we do recommend that future studies on relationships after ABI also consider the potential role of social cognition impairments. Those with social cognition impairments experience problems in understanding the mental states of others and using this information to guide their own social behavior [7]. Interrelated abilities underlying social cognition are emotion recognition, theory of mind and empathy. Deficits in this area are common after brain injuries [8] and are highly likely to have a strong impact on romantic relationships. However, to the best of our knowledge, only two quantitative studies to date have explored the effect of such deficits [9,10]. Additionally, while the sample sizes in these studies were small (*n* = 9 and *n* = 20, respectively) and more research is needed, their results indicated that social cognition impairments may indeed play an important role in couple relationships following ABI. In addition, it is conceivable that social cognition impairments may function as a confounding variable in some of the relations investigated by Laratta et al. [1] as they have also been found to be related to, for instance, educational level [8] and religious practices [11].

In conclusion, Laratta et al. [1] address a highly relevant and understudied topic by investigating which demographic and clinical factors influence the quality of partner relationships following ABI. The study’s merit is, however, somewhat limited by the small sample size. Moreover, we recommend that future studies on factors related to relationship quality following ABI consider the role of social cognition impairments as these impairments are common, likely to affect relationships and understudied.

## References

[1] Laratta S., Giannotti L., Tonin P., Calabrò R.S., Cerasa A. (2021). Marital Stability and Quality of Couple Relationships after Acquired Brain Injury: A Two-Year Follow-Up Clinical Study. Healthcare.

[2] Spanier G.B. (1976). Measuring dyadic adjustment: New scales for assessing the quality of marriage and similar dyads. J. Marriage Fam..

[3] Holahan C.J., Moos R.H. (1983). The quality of social support: Measures of family and work relationships. Br. J. Clin. Psychol..

[4] Godwin E.E., Kreutzer J.S., Arango-Lasprilla J.C., Lehan T.J. (2011). Marriage after brain injury: Review, analysis, and research recommendations. J. Head Trauma Rehabil..

[5] Kieffer-Kristensen R., Teasdale T.W. (2011). Parental stress and marital relationships among patients with brain injury and their spouses. NeuroRehabilitation.

[6] Faul F., Erdfelder E., Lang A.G., Buchner A. (2007). G*Power 3: A flexible statistical power analysis program for the social, behavioral, and biomedical sciences. Behav. Res. Methods.

[7] McDonald S. (2013). Impairments in social cognition following severe traumatic brain injury. J. Int. Neuropsychol. Soc..

[8] Sensenbrenner B., Rouaud O., Graule-Petot A., Guillemin S., Piver A., Giroud M., Béjot Y., Jacquin-Piques A. (2020). High prevalence of social cognition disorders and mild cognitive impairment long term after stroke. Alzheimer Dis. Assoc. Disord..

[9] Blonder L.X., Pettigrew L.C., Kryscio R.J. (2012). Emotion recognition and marital satisfaction in stroke. J. Clin. Exp. Neuropsychol..

[10] Burridge A.C., Huw Williams W., Yates P.J., Harris A., Ward C. (2007). Spousal relationship satisfaction following acquired brain injury. Neuropsychol. Rehabil..

[11] Schjoedt U., Stødkilde-Jørgensen H., Geertz A.W., Roepstorff A. (2009). Highly religious participants recruit areas of social cognition in personal prayer. Soc. Cogn. Affect. Neurosci..

